# Glial cells improve Parkinson’s disease by modulating neuronal function and regulating neuronal ferroptosis

**DOI:** 10.3389/fcell.2024.1510897

**Published:** 2025-01-03

**Authors:** Mengzhu Li, Mengxuan Chen, Haiyan Li, Da Gao, Lijun Zhao, Meiling Zhu

**Affiliations:** ^1^ The Fourth Clinical Medical College of Guangzhou University of Chinese Medicine, Shenzhen, Guangdong, China; ^2^ Shenzhen Clinical College of Integrated Chinese and Western Medicine, Guangzhou University of Chinese Medicine, Shenzhen, China

**Keywords:** Parkinson’s disease, ferroptosis, glia-neuron interaction, dopaminergic neuron, glial cell, microglia, astrocyte, oligodendrocyte

## Abstract

The main characteristics of Parkinson’s disease (PD) are the loss of dopaminergic (DA) neurons and abnormal aggregation of cytosolic proteins. However, the exact pathogenesis of PD remains unclear, with ferroptosis emerging as one of the key factors driven by iron accumulation and lipid peroxidation. Glial cells, including microglia, astrocytes, and oligodendrocytes, serve as supportive cells in the central nervous system (CNS), but their abnormal activation can lead to DA neuron death and ferroptosis. This paper explores the interactions between glial cells and DA neurons, reviews the changes in glial cells during the pathological process of PD, and reports on how glial cells regulate ferroptosis in PD through iron homeostasis and lipid peroxidation. This opens up a new pathway for basic research and therapeutic strategies in Parkinson’s disease.

## 1 Background

Parkinson’s disease (PD) is a common neurodegenerative disease that usually occurs in the elderly. The main manifestations are motor dysfunction, such as tremors, muscle stiffness, and bradykinesia. The main pathological features of PD are the loss of dopaminergic neurons (DA neurons) in the substantia nigra and the formation of Lewy bodies (LB). The underlying disease mechanisms include immune activation, mitochondrial dysfunction, lipid homeostasis disorder, and metal ion imbalance (Tolosa et al., 2006; Jankovic, 2008; Hayes, 2019). However, the etiology and pathological mechanism of PD remain unclear. Ferroptosis is a newly discovered way of cell death related to iron metabolism disorder. Iron is an indispensable element in life activities and participates in many biological processes, such as DNA synthesis, energy metabolism, and signal transduction. This unique cell death mechanism, driven by iron-dependent phospholipid peroxidation, is regulated by various cellular metabolic pathways. These pathways include redox homeostasis, iron metabolism, lipid and sugar metabolism, mitochondrial activity, amino acids, and various disease-related signaling pathways (Stockwell et al., 2017; Yan et al., 2021). In recent years, lots of evidence showed that the iron content in the substantia nigra of PD patients increased, suggesting that ferroptosis may be related to the pathogenesis of PD (Depierreux et al., 2021; Biondetti et al., 2021).

Glial cells are the main components of the central nervous system, including astrocytes, microglia, and oligodendrocytes. The interaction between glial cells and neurons is crucial for maintaining the normal function of the nervous system. For example, glial cells can provide nutritional support, participate in neurotransmitters’ recovery and metabolism, and regulate neurons’ survival and death. Other studies have confirmed that glial cell activation plays an important role in the onset and progression of PD (Fernández-Mendívil et al., 2021; Zhu et al., 2017). Activated glial cells can be neuroprotective by releasing neurotrophic factors and phagocytosis.

On the contrary, they can also mediate neuronal damage by releasing proinflammatory cytokines (Qian et al., 2023; Wang ZL. et al., 2022a). Activated glial cells promote iron homeostasis disorder, which aggravates iron toxicity of DA neurons (Riederer et al., 2023). Therefore, studying the interaction between glial cells and neurons, especially in ferroptosis, is of great significance for understanding the pathological mechanism of PD and developing new treatment strategies.

To decipher the regulatory mechanisms of ferroptosis in PD, particularly the role of neuroglial cells, we focused on dopamine (DA) production, transport processes, and neuronal excitability. Subsequently, we discussed alterations in different glial cells during the pathological progression and their roles in the development of PD. Finally, we reported how glia-neuron interactions regulate the process of ferroptosis in DA neurons through iron homeostasis and lipid peroxidation.

### 1.1 Interaction mechanism between glial cells and DA neurons

#### 1.1.1 Regulation of glial cells on dopamine

DA is the most abundant catecholamine neurotransmitter in the brain, which regulates various physiological functions of the central nervous system. Dopamine is synthesized by nerve cells or synapses themselves. In addition to being the precursor of norepinephrine, it is also an important neurotransmitter for maintaining the function of extrapyramidal nerves (Ehringer and Hornykiewicz, 1998). Many studies have shown that dopamine is important in regulating motor function, cognitive function, drug addiction, and slowing brain aging (Jankovic, 2008; Juarez and Samanez-Larkin, 2019; Liu and McNally, 2021).

In the central nervous system, the main synthesis pathway of DA is from L-tyrosine, which is transformed into L-dopa by tyrosine hydroxylase (TH) and then decarboxylated by aromatic acid decarboxylase (AADC) in the iron and H4-biopterin dependent pathway to produce DA. Glial cells regulate the activity by releasing extracellular signal molecules, such as glutamate and glial cell-derived neurotrophic factor (GDNF), thus affecting dopamine synthesis (Daubner et al., 2011; Montioli et al., 2013; Arnold and Salvatore, 2016; Chen et al., 2005; Salvatore et al., 2009). The activated oligodendrocyte precursor neuron/glial 2 (NG2) can be detected near TH-positive neurons in substantia nigra (SN) of PD model mice (Kitamura et al., 2010). Astrocytes can ingest and store dopamine from the extracellular fluid and transport dopamine to the endosomes through Vascular Monoamine Transporter 2 (VMAT2), actively releasing dopamine (Petrelli et al., 2020; Petrelli et al., 2023). VMAT2 and TH interact to protect neurons (Emborg et al., 2008; Weihe et al., 2006). Glial cells’ dopamine transporter (DAT) not only participates in the release of DA but is also responsible for reuptaking DA from the extracellular space into the cell (Monzani et al., 2019). In addition, flavin adenine dinucleotide (FAD) dependent monoamine oxidase (MAO) can promote the degradation of DA (Yu et al., 2016).

In summary, glial cells maintain DA levels by regulating DA’s synthesis, uptake, and release processes, thereby playing an important regulatory role in signal transmission between neurons and neurological function.

#### 1.1.2 The regulatory effect of glial cells on neuronal excitability

Neuronal hyperexcitability is considered an important mechanism in neurodegenerative diseases represented by PD. When the extracellular potassium concentration increases due to neuronal activity, astrocytes can sense and respond and help maintain the appropriate level of excitatory activity by regulating the distribution of ions. However, in traumatic brain injury, infection, and other diseases, resting astrocytes can transform into abnormal reactive astrocytes, causing neuronal excitability toxicity and leading to neuronal death (Qian et al., 2023; Guttenplan et al., 2021). Microglia make direct contact with neuronal synapses through stretching and retracting processes, thereby “monitoring” abnormal neuronal discharge and preventing the onset of neurological diseases (Merlini et al., 2021). Abnormally activated microglia are important factors triggering neuronal excitatory toxicity (Strackeljan et al., 2021). However, microglia are highly dynamic under physiological and pathophysiological conditions. Recent studies have shown that neuronal excitation can also rapidly recruit microglia activation (Eyo et al., 2016; Eyo et al., 2014). In addition, oligodendrocytes produce myelin, which provides insulation for axons and accelerates neuronal transmission (Lajoso et al., 2021).

Glial cells regulate the activity of DA neurons by releasing and reuptaking the neurotransmitter glutamate. Glutamate is the most abundant neurotransmitter in the central nervous system, and extracellular glutamate receptors can be found at the synaptic site of neurons, astrocytes, oligodendrocytes, or microglia. Their activation leads to varying amplitude and dynamic changes in neuronal excitability, promoting ATP release and preventing neuronal overexcitement. JWA deficiency in astrocytes can exacerbate dopaminergic neurodegeneration by reducing glutamate transporters (Wang et al., 2018). A recent study has found that specialized astrocytes in the adult brain, similar to glutamatergic synapses, can rapidly release glutamate under stimulation, maintaining long-term potentiation of neural processes (de Ceglia et al., 2023). Inhibition of SGK1 can enhance glial cell activity to clear glutamate toxicity and protect DA neurons. It has been reported as a key pathological feature and therapeutic target of Parkinson’s disease in recent years (Kwon et al., 2021). In addition, abnormal glial cells may also lead to changes in the extracellular environment, such as an increase in extracellular pH or disruption of ion balance, thereby affecting the normal function of DA neurons (Wang et al., 2003; Gibson et al., 2018; Teismann et al., 2003). At the same time, neurons have a supportive and complementary effect on the specific molecular developmental regulation of glial participating layers (Xie et al., 2022).

### 1.2 Neurons regulate the biological functions of glial cells

Neurons profoundly influence the glial cell system, playing a crucial role in the normal development, functional maintenance, and disease processes of the nervous system. Neuronal activity can regulate the proliferation and differentiation of glial precursor cells, particularly oligodendrocytes and astrocytes. Neurons trigger structural remodeling and morphological changes in astrocytes by releasing neurotransmitters, such as GABA, which bind to receptors on the surface of astrocytes (Taylor and Monje, 2023). Neuronal activity further modulates glial cell functions. After neurons release neurotransmitters into the synaptic cleft, astrocytes swiftly uptake these neurotransmitters through transporters on their surface, preventing their excessive accumulation and thus averting hyperstimulation of neurons. Astrocytes express high-affinity glutamate transporters, such as GLT-1 and GLAST, which efficiently clear glutamate from the synaptic cleft, a process vital for maintaining the normal function of glutamatergic synapses. Under specific conditions, astrocytes can also release neurotransmitters like D-serine into the synaptic cleft, thereby regulating the function of neuronal N-methyl-D-aspartate (NMDA) receptors and influencing the strength and direction of neurotransmission (Cheng et al., 2023). In neurological diseases, neuronal injury and degeneration lead to the activation of neuroglial cells and the exacerbation of inflammatory responses. In Parkinson’s disease, the degeneration of dopaminergic neurons results in the activation of microglia in the substantia nigra and the initiation of inflammatory reactions, which further exacerbate neuronal damage, creating a vicious cycle that accelerates the progression of the disease (Mancuso et al., 2024; Sacks et al., 2018).

### 1.3 “Transdifferentiation” of glial cells towards neurons

Neuron loss has always been a significant problem faced by various neurodegenerative diseases and injuries. Glial cells are a large and heterogeneous population of nerve cells almost evenly distributed in the brain and spinal cord parenchyma. Therefore, as a natural endogenous cell bank, how to use the physiological characteristics of glial cells to initiate the potential neural regeneration process has become the focus of many researchers. In recent years, scientists have achieved the induction of neuronal regeneration in mammals through gene editing, which provides a good foundation for the treatment of diseases related to neuronal loss (Jorstad et al., 2017; Zamboni et al., 2020). Professor Chen Gong’s team confirmed that overexpression of the NeuroD1 gene through the AAV virus vector can transdifferentiate astrocytes in the brain into neurons (Guo et al., 2014; Chen et al., 2020). In addition, some studies have confirmed that the knockdown of the PTBP1 gene can also realize the transdifferentiation of astrocytes into neurons (Qian et al., 2020; Zhou et al., 2020). The morphology and function of these induced neurons are highly consistent with those of endogenous neurons and have significant therapeutic effects on PD, Huntington’s disease (HD), and other diseases. However, many scholars have questioned the repeatability and preciseness of the results (Hoang et al., 2023; Wang LL. et al., 2021a; Rao et al., 2021). Several recent studies have provided direct evidence, such as two-photon *in vivo* imaging, to show the pedigree relationship between reprogrammed glial cells and newborn neurons (Liang et al. 2022; Jiang et al., 2021; Gao et al., 2009). Overall, the neural *in situ* transdifferentiation technology is still in the exploratory stage, and more exploration is needed in the future to provide more treatments for neurodegenerative diseases.

## 2 Glial cell dysfunction in Parkinson's disease

Human brain single-cell sequencing reveals glial cell activation and Parkinson-specific neuronal status. Pet of PD patients showed that whole brain microglia activation can induce the production of neurotoxic reactive astrocytes, which in turn will interfere with the survival of oligodendrocytes. Glial cell pathology drives the PD process associated with neuronal death throughout the brain (Smajić et al., 2022).

### 2.1 Morphological and functional changes of glial cells in PD patients

#### 2.1.1 Oligodendrocytes

In the central nervous system, oligodendrocytes are mainly responsible for producing and maintaining the myelin sheath of neurons. The structural integrity of the myelin sheath is the basis for the transmission of action potential along myelinated axons. In PD patients, abnormal oligodendrocyte function can lead to the loss of myelin sheath. At the same time, reducing the conduction velocity and efficiency of nerve signals directly affects the functions of human motion control, balance, and coordination. Oligodendrocytes are the cells with the most iron content in the central nervous system. Therefore, under pathological conditions such as demyelination, oligodendrocytes may release a large amount of iron and damage neurons. According to the gene expression data of the brain of dead patients at different disease stages, the expression of PD-related genes in oligodendrocytes has increased in the early stage of PD, even earlier than the degeneration of DA neurons in the substantia nigra, and the generation of motor symptoms (Lake et al., 2016; Lake et al., 2018). Experiments have been carried out to use neuronal and oligodendrocyte marker secretory bodies and detect α-syn. Oligodendrocytes and neuronal exosomes α- The ratio between syn concentrations as a biomarker to distinguish PD (Dutta et al., 2021). In addition, oligodendrocytes provide an antioxidant defense system for neurons by secreting FTH1 to resist iron-induced cytotoxicity (Dang et al., 2022).

Due to the increase in cell death and the decrease in cell regeneration, the number of oligodendrocytes in the brains of patients with Parkinson’s disease decreased significantly (Fan et al., 2023). The decrease of oligodendrocytes and functional damage can lead to neurodegenerative changes. This can lead to the death of DA neurons in the brain, leading to the typical symptoms of Parkinson’s disease (Yang et al., 2023).

#### 2.1.2 Microglia

As immune cells in the central nervous system, microglia have dynamic morphology and high plasticity (Savage et al., 2019). Microglia were distributed unevenly, especially in the midbrain and substantia nigra, which were closely related to the pathology of Parkinson’s disease (Smajić et al., 2022). Microglia are highly sensitive and easy to activate. In the resting state, they are branched, and the activated microglia are amebic-like. These cells are round, with pseudopodia and fibrillary processes, and contain many lysosomes (Tsukahara et al., 2020). Activated microglia can reach the injured site and release neurotoxic factors such as prostaglandins, reactive oxygen species (ROS), and proinflammatory cytokines. This leads to neuroinflammatory reactions and neuronal damage, triggering neuronal apoptosis cascade reactions (Tansey and Goldberg, 2010; Giordana et al., 1994; Dihné et al., 2001; Sun et al., 2018; Maurya et al., 2021). The activation of microglia was found in the PD model intervened by 1-Methyl-4-phenyl-1,2,3,6-tetrahydropyridine (MPTP), lipopolysaccharide (LPS), and rotenone (Lee et al., 2019; Machado et al., 2016; Zhang et al., 2021; Zhou et al., 2023). Neuron loss in PD will release abnormal aggregates of α-synuclein (α-syn), which microglia will absorb through CX3CL1/CX3CR1 and other pathways. Microglia will release various inflammatory mediators, leading to an increase in inflammatory response (Wang et al., 2020). These inflammatory mediators will further activate other immune cells, such as macrophages and T cells, and form an inflammatory environment. This inflammatory environment will lead to the injury and death of neurons and further aggravate the pathological process of Parkinson’s disease. Additionally, α-syn can also inhibit autophagy in microglia. Microglia jointly degrade fibrillar α-syn cargo by distribution through tunneling nanotubes, slowing down PD progression (Scheiblich et al., 2021; Tu et al., 2021).

#### 2.1.3 Astrocytes

Usually, the cell body of astrocytes has multiple protrusions that can interconnect with neurons and provide support and nutrition. The cell bodies of PD patients are swollen, and the cell processes are reduced or more complex. DA deprivation significantly caused the activation and proliferative changes of astroglial fibrillary acidic protein-positive cells in the striatum (Zhu et al., 2020). In addition, astrocytes also accumulate abnormally aggregated proteins in the cytoplasm, such as α-syn (Wang C. et al., 2021b). The morphological changes of astrocytes and the accumulation of abnormal proteins in Parkinson’s disease may cause inflammatory responses and neuronal damage. Glial cultures differentiated from iPSCs from patients with PARK2-related Parkinson’s disease showed doubled expression of genes encoding inflammatory cytokines (Gerasimova et al., 2023). The abnormal activation and inflammatory response of astrocytes may release some harmful substances, such as ROS, to further damage the surrounding neurons (Emerit et al., 2004; Hass and Barnstable, 2016). In addition, astrocytes’ abnormal morphology and function may affect neurons’ normal functions, such as synaptic transmission and neurotransmitter release (Chen et al., 2023; Iovino et al., 2020). Extracellular vesicles released by activated astrocytes EV mediate or aggravate the pathological process of PD (Upadhya et al., 2020).

Glial cells also interact with each other in Parkinson’s disease models. Activating microglia induces neurotoxic reactive astrocytes microglia block A1 astrocyte transformation (Liddelow et al., 2017; Yun et al., 2018). ROS can also trigger the phenotypic switch between astrocytes and microglia (Hass and Barnstable, 2016).

In conclusion, glial cells’ morphological and functional changes may be related to PD’s pathological process and neuronal damage. Further study of these changes will help to understand the pathogenesis of PD and find new therapeutic methods.

### 2.2 Role of glial cells in inflammatory response and oxidative stress in Parkinson’s disease

Neuroinflammation plays an important role in the pathogenesis of PD. Both central nervous and systemic inflammation can lead to degeneration and death of DA neurons. Many mutational factors that cause PD, including α-syn, Leucine-rich repeat kinase 2 (LRRK2), Glucocerebrosidase (GBA), Parkin RBR E3 Ubiquitin Protein Ligase (PRKN), Parkin RBR E3 Ubiquitin Protein Ligase (PINK1), and Parkin RBR E3 Ubiquitin Protein Ligase (DJ-1), etc. They are all associated with intestinal and neuroinflammation (Keshavarzian et al., 2015; Wang L. et al., 2022b). In PD, inflammatory cells such as macrophages and CD4 T cells release inflammatory mediators (Williams et al., 2021). Researchers found that proinflammatory factors such as interleukin-1β (IL-1β), interleukin-2 (IL-2), interleukin-6 (IL-6), interleukin-10 (IL-10), and tumor necrosis factor-α (TNF-α) can be used as important inflammatory biomarkers to detect PD from the serum and cerebrospinal fluid (CSF) of patients with PD (Liu et al., 2022). In the PD model, α-syn accumulation and the signals transmitted by toll-like receptors (TLRs) lead to the M1 proinflammatory phenotype of microglia and activate the NOD-, LRR- and pyrin domain-containing protein 3 (NLRP3) inflammasome. The permeability of the blood-brain barrier (BBB) is changed, and brain infiltration is induced by circulating leukocytes to enhance the inflammatory response of the nervous system (Wang WY. et al., 2015a; Lv et al., 2023; Han et al., 2019; Salvi et al., 2017; Na et al., 2010; Soraci et al., 2023). The activation of microglia and astrocytes under inflammatory conditions in mouse models of Parkinson’s disease is region-dependent, especially in the striatum and midbrain (Basurco et al., 2023). Oligodendrocytes and astrocytes also participate in releasing inflammatory mediators and the repair process after neuronal injury. These mediators can further activate other glial cells and immune cells, forming a positive feedback loop of inflammatory response. However, the overreaction of glial cells may also lead to excessive inflammatory response, which may harm neurons.

Inflammatory reactions can also lead to oxidative stress, that is, excessive production of oxygen free radicals in cells, which exceeds the ability of cells to scavenge. Activated glial cells can lead to oxidative stress, and excessive oxygen free radicals damage the cell membrane, mitochondria, and DNA, thus exacerbating the inflammatory response and even leading to neuronal damage and death. P2X purinoceptor 7 (P2X7) receptors widely exist in microglia, astrocytes, and oligodendrocytes, and they regulate the neuronal function of the central nervous system of PD by releasing proinflammatory cytokines/chemokines, ROS, and the excitotoxic gliotransmitters glutamate and ATP. Damp-induced NADPH oxidase (NOx), which encodes NADPH oxidase, is the primary source of ROS production in activated microglia, can produce ROS and nitric oxide through Nitric Oxide Synthase 1 (NOS1) and Nitric Oxide Synthase 3 (NOS3), maintain chronic inflammation and cause neuronal death (Chen et al., 2016). Histamine activates microglial activation and dopaminergic neuronal toxicity through Histamine H1 Receptor (H1R) and triggers ROS production through H1R and Histamine H4 Receptor (H4R) activation (Rocha et al., 2016). Oxidative stress caused by microglia activation also affects autophagy and mitochondrial function in neurons. Microglial protein kinase C delta (PKCδ) elevates neuroinflammatory responses, while inhibiting PKCδ activation reduces neuronal loss (Gordon et al., 2016). In animal and cellular models of Parkinson’s disease, microglial mitochondrial damage amplifies the Nucleotide-binding oligomerization domain, Leucine-rich Repeat, and Pyrin domain-containing Protein 3 (NLRP3) inflammasome proinflammatory signals. It causes neuronal death while activating parkin-mediated mitophagy can inhibit NLRP3 inflammasome activation in microglia and protect neurons (Sarkar et al., 2017; Ahmed et al., 2021).

Reactive astrocytes can release various antioxidant molecules, such as metallothionein (MT) and ROS-activating transcription factor Nuclear factor erythroid 2-related factor 2 (Nrf2), which are key neuroprotective regulators of oxidative stress, to reduce neuronal toxicity caused by oxidative stress. In addition, in PD, astrocytes produce a large number of antioxidant enzymes, such as superoxide dismutase (SOD) and glutathione peroxidase (GPX), to scavenge reactive oxygen species and alleviate oxidative stress. However, when oxidative stress is excessive, the antioxidant capacity of astrocytes may be damaged, thereby aggravating neuronal damage. Oxidative stress and inflammatory reaction will damage oligodendrocytes to a certain extent, destroying the nerve myelin sheath and further affecting nerve conduction (Spaas et al., 2021). α-syn misfolded proliferation from neurons to oligodendrocytes and spread between cells in a “prion-like” manner will cause oxidative stress, proteasome and mitochondrial dysfunction, and dysregulation of myelin lipids (Jellinger, 2018).

In general, glial cells play an important role in oxidative stress in PD. Oxidative stress caused by inflammation can activate microglia, astrocytes, and oligodendrocytes, resulting in their release of inflammatory mediators and oxygen-free radicals, which in turn damage nerve cells and nerve fibers. Further study of these mechanisms and pathways will help to understand the pathogenesis of Parkinson’s disease and provide a theoretical basis for developing new therapeutic strategies.

### 2.3 Influence of glial-related factors on PD

In addition to inflammatory factors, many neurotrophic factors are associated with glial cells in Parkinson’s disease. Glutathione (GSH) is an antioxidant that plays a protective role in neurons. The level of intracellular GSH determines whether nitric oxide (NO) has a neurotrophic effect on DA neurons. Depletion of reduced glutathione occurs in substantia nigra and sporadic LB disease of PD, resulting in oxidative stress damage to neurons. MPP + -induced Transient Receptor Potential Cation Channel Subfamily M Member 2 (TRPM2) channel activation in microglia is regulated by glutathione and plays a central role in oxidative cytotoxicity and inflammation (Yıldızhan and Nazıroğlu, 2020). Cystathionine β- Synthase (CBS) is a common precursor of GSH and other sulfur molecules. The destruction of the CBS/H2S signal in microglia of the PD mouse model is easily affected by LPS-induced activation of NLRP3 inflammasome and loss of DA neurons (Mou et al., 2023). Protease-activated receptor-1 (PAR-1) is only expressed in glial fibrillary acidic protein-positive astrocytes, and the number of astrocytes expressing PAR-1 in PD SNpc increases. The expression of glial cell line-derived growth factors and GPX increased, but the expression of nerve growth factors and inflammatory cytokines such as IL-8 and MCP-1 did not change. The present study suggests that increased expression of PAR-1 in astrocytes in Parkinson’s disease brain regions is beneficial for neuroprotection in the pathological process of PD (Ishida et al., 2006). Extracellular signal-regulated protein kinase (ERK) activation occurs only in glial cells, mainly astrocytes, and less frequently in oligodendrocytes and glial progenitor cells. Due to the continuous activation of the ERK-1/2 pathway in glial cells, after GSH depletion, the neurotrophic properties do not turn into neurotoxicity, which aggravates PD pathology (Canals et al., 2003).

Fibronectin is a macromolecular glycoprotein that widely exists in the extracellular matrix and regulates the synaptic transmission of DA neurotransmitters. Fibronectin exists in all types of glial cells. Fibronectin can interact with integrin to promote the activation and migration of glial cells, thus participating in neuroinflammation and the growth and development of neurons (Rieske et al., 2009; Neiiendam et al., 2004; Wang et al., 2013). Fibronectin in glial cells can interact with α-syn interacts to form fibronectin in glial cells- α- Synuclein complex. The formation of this complex may lead to the abnormal aggregation of α-syn, forming the characteristic body of PD (Van Gool et al., 1994; Tsuboi et al., 2005). Fibronectin can also affect the immune response. Fibronectin can regulate the activation and function of immune cells by interacting with receptors on glial cells, such as Cluster of Differentiation 44 (CD44) and Purinergic Receptor P2Y2 (P2Y2), thereby affecting neuroinflammation and neuroprotection (Abbaszadeh et al., 2014; Chorna et al., 2004). Therefore, fibronectin may interact with glial cells through the above ways to participate in the pathogenesis of PD. However, fibronectin’s exact role and mechanism in PD need further study.

## 3 The role of glial cells in ferroptosis during Parkinson's disease

### 3.1 Mechanisms underlying ferroptosis-induced Parkinson’s disease

Iron is essential for cell development and maintaining various physiological processes in the central nervous system. Abnormal iron deposition in the brain leads to neurotoxicity by producing free radicals and oxidative stress. Ferroptosis is a new type of iron-dependent cell regulatory death. Ferroptosis is an iron-ion-dependent novel form of regulated cell death whose mechanism primarily involves iron transport, amino acid metabolism, and lipid peroxidation. Under the influence of ferrous ions or lipoxygenases, the overexpressed unsaturated fatty acids on the cell membrane undergo lipid peroxidation, thereby inducing cell death. This process is accompanied by decreased expression levels of antioxidant systems, such as glutathione (GSH) and glutathione peroxidase 4 (GPX4). Research indicates that Parkinson’s-related cognitive impairment is closely associated with ferroptosis. The accumulation of Lewy bodies, formed by the deposition of α-synuclein (α-syn), is the core pathological mechanism underlying Parkinson’s-related cognitive impairment. Iron ions, particularly ferrous ions (Fe^2^⁺), can catalyze the generation of reactive oxygen species (ROS) through the Fenton reaction, thereby initiating an oxidative stress state. This oxidative stress not only directly damages lipids, proteins, and nucleic acids within cells but also promotes conformational changes in α-syn, transitioning it from a natively disordered state to an ordered β-sheet structure through mechanisms such as phase separation, leading to the aggregation of α-syn (Li et al., 2022).

Furthermore, α-syn possesses the ability to bind with iron ions. α-Syn phosphorylation and oxidative dysregulation mediate iron and dopamine-dependent oxidative stress through impaired cellular localization. These post-translational modifications enhance the aggregation propensity of α-syn and promote iron reduction cycle, forming a self-amplifying positive feedback loop (Duce et al., 2017).

### 3.2 Regulation of glial cells on iron metabolism in PD

Iron toxicity has become an important pathogenesis of Parkinson’s disease. The glial cytoplasmic inclusions (GCI) in oligodendrocytes are formed by misfolded α-syn and contain a large amount of iron, which is closely related to the neurodegeneration of cerebellum, striatum, and SN (Kaindlstorfer et al., 2018). Astrocytes mediate the transport of various types of iron by binding to ceruloplasmin (CP) and interacting with transferrins such as Transferrin (TF), Transferrin Receptor (TFR), Ferroportin (FPN), and Divalent Metal Transporter 1 (DMT1), and then promote the transferrin synthesized by oligodendrocytes in the brain to bind to most of the iron through the BBB)after iron oxidation (Rouault and Cooperman, 2006; Dringen et al., 2007; Xu et al., 2017; Jeong and David, 2003; Beydoun et al., 2017). Studies have shown that the loss of CP and subsequent iron accumulation in the SN, MPTP-induced PD mouse model, and 6-OHDA injured mouse model of PD patients exacerbate the death of DA neurons in PD (Youdim and Riederer, 1993; Friedrich et al., 2021; Ayton et al., 2013; You et al., 2015; Wang J. et al., 2015a). Iron homeostasis in microglia can be regulated by angiotensin, thereby improving the progress of microglial inflammatory response and dopaminergic degeneration. Ginsenoside Rg1 can maintain iron-regulated protein homeostasis by increasing the expression of ferritin heavy chain and decreasing the expression of ferritin light chain (FTL) in natural oligodendrocytes (OLs) to resist lipid peroxidation stress in oligodendrocytes, thus playing a neuroprotective role (Chen et al., 2022). Glial cells play an important role in the effective regulation of iron homeostasis in DA neurons (Garrido-Gil et al., 2013).

Glial cells can store iron ions and release them when needed. In PD, glial cells may maintain the balance of iron ions by regulating the storage and release of iron ions to protect neurons from the toxic effects of iron ions. Ferritin is the major iron storage protein and accounts for most of the iron in the brain. Ferritin is mainly present in oligodendrocytes. The different mineralization of iron inside ferritin may be related to oxidative stress in oligodendrocytes, which could affect myelination processes with the consequent perturbation of information transference (Quintana and Gutiérrez, 2010). Astrocytes may secrete ferritin through Transient Receptor Potential Mucolipin 1 (TRPML1) - mediated exocytosis (Yu et al., 2023). In MPP + -induced MES23.5 dopaminergic cells, astrocytes increase ferritin release in response to iron overload to protect DA neurons (Zhang et al., 2022). In substantia nigra, the activation of microglia and the loss of reactive astrocytes in PD were confirmed by immunohistochemical detection of cr3\/43 and ferritin (Mirza et al., 2000). Heme oxygenase-1 (HO-1) - mediated massive autophagy can lead to unregulated iron deposition in PD. The transmembrane iron chelator desferridone (DFP) can improve the overexpression of HO-1 in astrocytes and lead to iron deposition in the striatum (Song et al., 2017; Zukor et al., 2009).

In general, glial cells maintain the balance of iron ions in PD from the aspects of iron uptake, transport, and storage to protect DA neurons from the toxic effects of iron ions. This regulatory effect may play an important role in the occurrence and development of PD.

### 3.3 Regulation of glial cells on lipid peroxidation in PD

Mechanistically, ferroptosis is an iron-dependent, highly expressed unsaturated fatty acid on the cell membrane that undergoes lipid peroxidation. Iron ions play an important catalytic role in lipid peroxidation. Iron is a transition metal with a strong conversion ability, which can carry out redox reactions between fe^2+^ and fe^3+^. In this process, iron can catalyze the generation of many reactive oxygen species, such as superoxide anion (O2-), hydroxyl radicals (•OH), etc. These reactive oxygen species have strong oxidizing properties. They can attack the lipids on the cell membrane, leading to lipid peroxidation, which in turn causes the destruction of the structure and function of the cell membrane and ultimately leads to neuronal death. In addition, in PD, the increased activity of NOx in microglia, the decreased activity of glutathione peroxidase in oligodendrocytes, the decreased activity of SOD in astrocytes, and the abnormal metabolism of glutathione all lead to the accumulation of intracellular superoxide, which in turn increases the occurrence of lipid peroxidation. Paraquat (PQT) is used as an animal model of PD to induce peripheral nerve demyelinating neuropathy by enhancing ROS production in oligodendrocytes and regulating Liver X Receptor (LXR) and Wingless/Integrated (WNT) pathways (Hichor et al., 2017). Glitazones (G1 and G2), through the agonistic activation of PPAR-γ, stimulate the PGC-1α signaling in the brain, restoring the levels of antioxidant enzymes glutathione and superoxide, activating pyramidal neurons, increasing the number of oligodendrocytes, and reducing the lipid peroxidation intensity in brain tissues. Promising results have been demonstrated in the treatment of PD (Prabhakaran et al., 2023). Studies have found that palmitoylethanolamide (PEA) can promote neurogenesis and inhibit astrocyte activation. It also demonstrated to mitigate β-amyloid-induced astrogliosis by modulating lipid peroxidation, reactive oxygen species production, caspase3 activation, etc (Colizzi et al., 2022). After glial cell activation, the nuclear transcription factor Nuclear Factor-kappa B (NF- κ B) is activated, which upregulates the expression of inflammation and oxidative stress genes. Glia matery factor (GMF) deficiency can inhibit NF- κ B activity, reducing astrocyte oxidative stress (Khan et al., 2014).

In conclusion, glial cells are important in regulating iron metabolism and lipid peroxidation in Parkinson’s disease. Exploring the role of glial cells in ferroptosis may suggest a new strategy to inhibit ferroptosis and regulate the process of Parkinson’s disease by focusing on the interaction between glial cells and DA neurons.

## 4 Discussion

**FIGURE 1:**
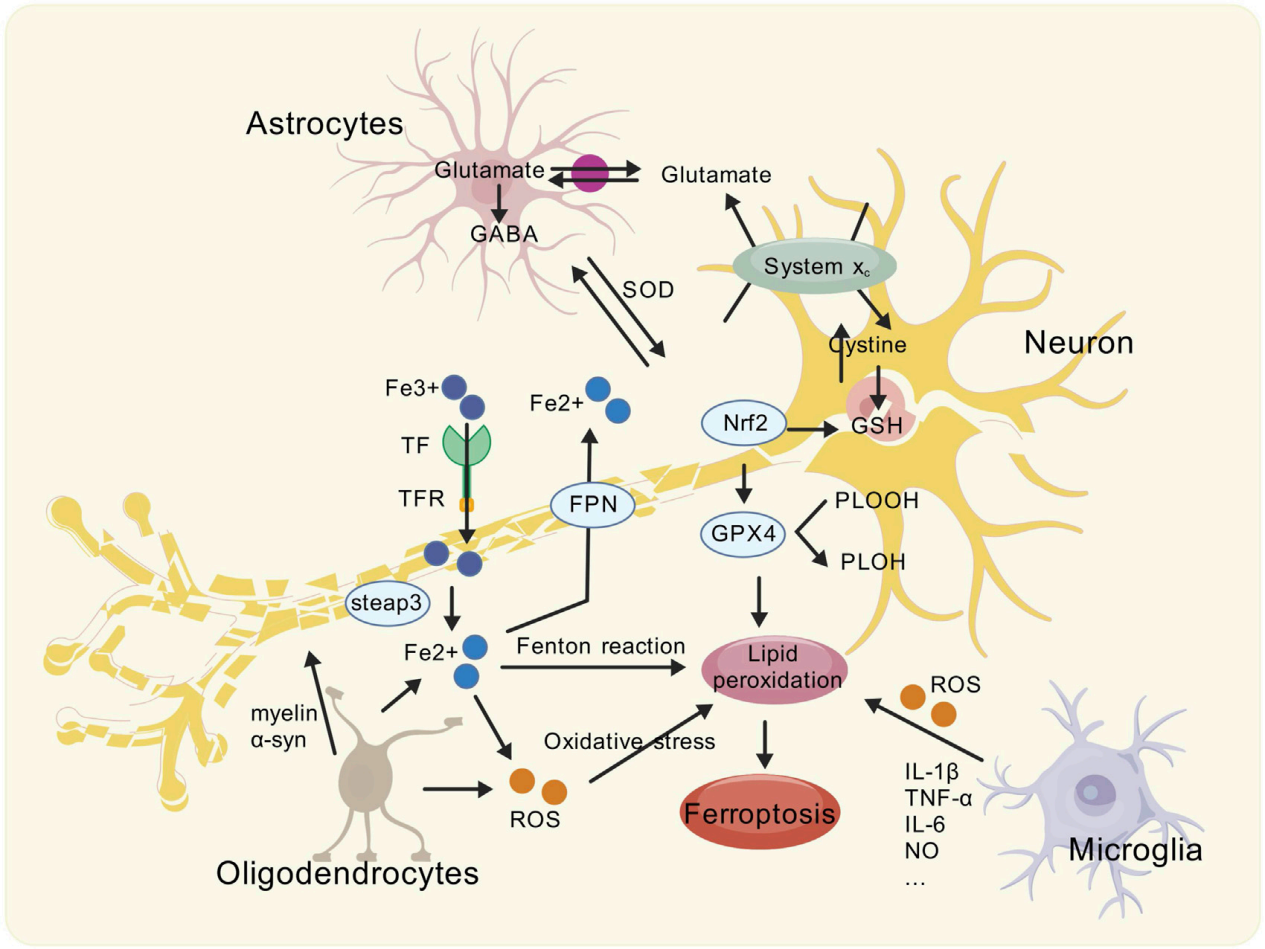
Schematic showing the modes of ferroptosis caused by Glia-neuron interactions in the PD brain. The brain of PD is associated with ferroptosis. Activated reactive astrocytes, microglia, and oligodendrocytes can undergo inflammatory reactions, causing oxidative stress, exacerbating neurotoxicity, and affecting the health of dopaminergic neurons. Meanwhile, abnormally activated glial cells further interact with neurons by affecting transferrin to exacerbate iron deposition and lipid peroxidation, ultimately leading to ferroptosis.

Parkinson’s disease is a chronic progressive neurological disease characterized by the death of DA neurons in the substantia nigra, resulting in symptoms such as dyskinesia, muscle stiffness, and tremor. Studies have confirmed that iron deposition in the substantia nigra is an important factor in the degeneration of DA neurons. Ferroptosis is an iron-dependent cell death, and its molecular mechanism is closely related to iron homeostasis, glutathione metabolism, and lipid metabolism. Glial cells are a class of non-neuronal cells in the central nervous system whose primary function is supporting and protecting neurons. Recent studies have shown that glial cell abnormalities may be an important mechanism leading to the death of DA neurons. Therefore, we speculate that glial cell interaction may be the key mechanism of ferroptosis in Parkinson’s disease (Figure 1).

Glial cells can regulate the synthesis, uptake, and release of DA and neuronal excitability. Glial cell abnormalities may cause DA neurons’ death through various pathways. First, abnormal glial cells may increase neuroinflammatory response and release many inflammatory factors, such as TNF-α, IL-1β and IL-6, etc. These inflammatory factors can directly damage DA neurons and lead to their death. Secondly, abnormal glial cells can increase the production of oxygen free radicals, which can cause oxidative stress. Oxidative stress can lead to oxidative damage of proteins, lipids, and nucleic acids in cells and then death of DA neurons. Mitochondria is an important organ in cells, which is mainly responsible for the production of intracellular energy.

The mitochondrial membrane is an important source of ROS in cells. A small amount of free iron in cells is enough to catalyze the oxidative decomposition of unsaturated membrane fatty acid. Therefore, the abnormality of glial cells may damage mitochondrial function, leading to the insufficient energy supply of DA neurons and eventually their death. Abnormal glial cells may also lead to the death of DA neurons through other mechanisms, such as abnormal synaptic transmission function of neurons, increased apoptosis of neurons, etc. At present, studies have found that there is “transdifferentiation” between glial cells and neurons, which provides a new perspective for the exploration of PD treatment. The death of DA neurons may cause glial cell abnormalities. The specific mechanisms involve DA reduction, glial cell activation, functional abnormalities, and quantitative changes. These abnormalities may further affect the function and structure of the nervous system.

Abnormal glial cells and death of DA neurons may cause ferroptosis. The death of DA neurons will lead to the release and accumulation of iron ions in cells, which can catalyze the generation of free radicals through the Fenton reaction so that the electron transfer is blocked, the intracellular calcium balance is disordered, the role of protease is strengthened, the membrane lipid peroxidation is enhanced, and finally lead to cell death and the occurrence and development of PD. In addition, ferroptosis can further aggravate the death of DA neurons and increase inflammatory response and oxidative stress.

Early diagnostic biomarkers for Parkinson’s disease primarily encompass specific cerebrospinal fluid and plasma biomolecules. Recent research has indicated that specific pathological molecules, such asα-Syn, when encapsulated within extracellular vesicles (EVs), may traverse the blood-brain barrier. Consequently, EVs containing specific microRNAs, such as c in plasma, can potentially serve as biomarkers for Parkinson’s disease (Yu et al., 2024). Moreover, iron deposition and its associated molecular mechanisms, including alterations in the expression of DJ-1 and SLC7A11 genes, as well as the production of lipid peroxides, are closely related to ferroptosis in Parkinson’s disease (Lind-Holm Mogensen et al., 2023; Kong et al., 2024). However, the sensitivity and specificity of these biomarkers still require further validation and optimization to better apply them in the early diagnosis and treatment of Parkinson’s disease. The therapeutic drugs for Parkinson’s disease primarily consist of levodopa, amantadine, and dopamine receptor agonists such as pramipexole, which are used to enhance dopaminergic effects. Auxiliary medications, including catechol-O-methyltransferase inhibitors (e.g., opicapone) to reduce levodopa metabolism, anticholinergics (e.g., benztropine) to inhibit the cholinergic system, and monoamine oxidase B inhibitors (e.g., selegiline) to decrease dopamine degradation, collectively constitute the pharmacological regimen for comprehensive treatment of Parkinson’s disease (Vijiaratnam et al., 2021).

Additionally, the deferoxamine (DFO) nanomedicine has been found to exhibit potential in modulating ferroptosis for the improvement of Parkinson’s disease (Lei et al., 2024). Current research has identified the reduced form of the non-canonical vitamin K cycle as a potent ferroptosis inhibitor, capable of protecting neurons from ferroptosis-induced damage by scavenging reactive oxygen species (Mishima et al., 2022). For the treatment of PD, in addition to protecting dopaminergic neurons, there is increasing interest in immunomodulatory therapy. Various immunosuppressants, such as Azathioprine, Sargramostim, and Glatiramer, are considered potential drugs for slowing the progression of PD. However, the specific mechanisms of action and efficacy of these drugs require further research and validation (Greenland et al., 2020; Olson et al., 2021; Kasindi et al., 2022). However, no studies have yet identified means to treat PD through intervention in glia-neuron interactions directly. Therefore, investigating the interactions between glial cells and dopaminergic DA neurons, as well as the mechanisms of ferroptosis, can help us better understand the pathogenesis of PD, identify methods to reduce iron accumulation or mitigate its cellular damage, and provide clues for the development of novel therapeutic strategies.

However, the complexity of glial-neuron interactions poses a significant obstacle, not only in terms of the intricate signaling networks between them but also due to the functional differences and interdependencies among various glial cell subtypes. Understanding the fine-tuned regulatory mechanisms of these interactions is crucial for precise intervention in ferroptosis. Secondly, the difficulty in modeling ferroptosis and its relationship with PD *in vivo* must be addressed. Current animal models, represented by 6-OHDA, MPTP, and others, often need help to fully replicate the complexity of human disease, particularly in simulating long-term iron overload and the dynamic changes in glia-neuron interactions. Therefore, developing animal models and *in vitro* three-dimensional culture systems that more closely mimic human pathophysiological processes is vital. Finally, translating laboratory research findings into clinical applications faces the challenge of the translational gap. This requires interdisciplinary collaboration, strengthening the close integration of basic research with clinical practice, optimizing clinical trial designs, and exploring innovative therapeutic approaches to accelerate the translation of research findings into practical applications.

In conclusion, the abnormality of glial cells in PD may be an important mechanism leading to the death of DA neurons. Abnormal glial cells can cause the death of DA neurons in various ways, including an increase in inflammatory response and oxidative stress, and ultimately lead to ferroptosis, further aggravating the progress of PD. For the treatment of Parkinson’s disease, in addition to the protection of DA neurons, we should also pay attention to the functional regulation of glial cells to delay the progress of the disease. Studying the interaction between glial cells and DA neurons and the mechanism of ferroptosis can help us better understand the pathogenesis of Parkinson’s disease, find ways to reduce iron accumulation or damage to cells, and provide clues for developing new treatment strategies.

## Glossary

PD: Parkinson’s disease
DA: dopamine
CNS: central nervous system
DA neurons: dopaminergic neurons
TH: tyrosine hydroxylase
AADC: aromatic acid decarboxylase
NG2: neuron/glial 2
SN: Substantia nigra
VMAT2: Vascular Monoamine Transporter 2
DA: dopamine transporter
FAD: flavin adenine dinucleotide
MAO: monoamine oxidase
HD: Huntington’s disease
ROS: oxygen species
MPTP: 1-Methyl-4-phenyl-1,2,3,6-tetrahydropyridine
LPS: lipopolysaccharide
α-syn: α-synuclein
LRRK2: Leucine-rich repeat kinase 2
GBA: Glucocerebrosidase
PRKN: Parkin RBR E3 Ubiquitin Protein Ligase
PINK1: Parkin RBR E3 Ubiquitin Protein Ligase
DJ-1: Parkinson Disease Protein 7
IL-1β: Interleukin-1β
IL-2: Interleukin-2
IL-6: Interleukin-6
IL-10: Interleukin-10
TNF-α: Tumor Necrosis Factor-α
CSF: cerebrospinal fluid
BBB: blood-brain barrier
NLRP3: NOD-, LRR- and pyrin domain-containing protein 3
P2X7: P2X purinoceptor 7
NOx: NADPH oxidase
NOS1: Nitric Oxide Synthase 1
NOS3: Nitric Oxide Synthase 3
H1R: Histamine H1 Receptor
H4R: Histamine H4 Receptor
PKC δ: protein kinase C delta
NLRP3: Nucleotide-binding oligomerization domain, Leucine rich Repeat and Pyrin domain-containing Protein 3
GSH: Glutathione
MT: metallothionein
Nrf2: Nuclear factor erythroid 2-related factor 2
SOD: superoxide dismutase
GPX: glutathione peroxidase
NO: nitric oxide
CBS: Cystathionine β- Synthase
PAR-1: Protease-activated receptor-1
ERK: Extracellular signal-regulated protein kinase
CD44: Cluster of Differentiation 44
P2Y2: Purinergic Receptor P2Y2
GCI: glial cytoplasmic inclusions
CP: Ceruloplasmin
TF: Transferrin
TFR: Transferrin Receptor
FPN: Ferroportin
DMT1: Divalent Metal Transporter 1
FTL: ferritin light chain
Ols: oligodendrocytes
TRPML1: transient receptor potential mucolipin 1
HO-1: Heme oxygenase-1
DFP: desferridone
PQT: Paraquat
LXR: Liver X Receptor
WNT: Wingless/Integrated
PPAR-γ: Peroxisome Proliferator-Activated Receptor-γ
PEA: palmitoylethanolamide
NF- κ B: Nuclear Factor-kappa B
O2-: Superoxide anion
•OH: hydroxyl radicals
